# Digital Case Manager—A Data-Driven Tool to Support Family Caregivers with Initial Guidance

**DOI:** 10.3390/ijerph20021215

**Published:** 2023-01-10

**Authors:** Paul Wunderlich, Frauke Wiegräbe, Helene Dörksen

**Affiliations:** inIT—Institute Industrial IT, OWL University of Applied Sciences and Arts, Campusallee 6, 32657 Lemgo, Germany

**Keywords:** machine learning, healthcare, case management, caring, multi-label classification

## Abstract

Due to the demographic aging of society, the demand for skilled caregiving is increasing. However, the already existing shortage of professional caregivers will exacerbate in the future. As a result, family caregivers must shoulder a heavier share of the care burden. To ease the burden and promote a better work-life balance, we developed the Digital Case Manager. This tool uses machine learning algorithms to learn the relationship between a care situation and the next care steps and helps family caregivers balance their professional and private lives so that they are able to continue caring for their family members without sacrificing their own jobs and personal ambitions. The data for the machine learning model are generated by means of a questionnaire based on professional assessment instruments. We implemented a proof-of-concept of the Digital Case Manager and initial tests show promising results. It offers a quick and easy-to-use tool for family caregivers in the early stages of a care situation.

## 1. Introduction

The aging population in Germany is resulting in a growing need for care. According to the Federal Statistical Office, 4.1 million people in Germany currently require care under the German Long-Term Care Insurance Act (SGB XI). Of these, approximately 80% are cared for at home [1]. It is anticipated that in the coming years, there will be an increase in chronic diseases such as stroke and dementia, leading to a higher demand for therapy and care. Research by Glaeske [2] indicates that the need for care is increasing and that a decline in cognitive performance is strongly correlated with a need for assistance.

In light of these developments, interactive technologies are becoming more popular as a means of providing medical and nursing care to those in need. According to the German Federal Ministry of Education and Research (BMBF2022), these technologies may significantly improve the quality of care by relieving the physical and mental strain that informal and formal caregivers experience in the course of their daily duties and by raising the standard of care given by the care profession. Instead of replacing trained caregivers, these technological tools are designed to complement and support them in their duties, lightening their workload and streamlining their job. This method considers both the people receiving care and the larger network of healthcare professionals. Glauner [3] sees great potential for the application of Artificial Intelligence (AI) in healthcare. Artificial Intelligence can enable more personalized care for patients and help reduce healthcare costs and waiting times. Currently, the application of Artificial Intelligence and machine learning in healthcare is largely concentrated on the diagnosis of diseases, such as diabetes [4,5], breast cancer [6], Alzheimer’s disease [7], kidney disease [8,9], thyroid disease [10], and many others. This is particularly true for diagnostic methods that provide efficient data for model learning, such as image-based procedures like magnetic resonance imaging (MRI) [11], computer tomography (CT) [12,13], positron emission tomography (PET) [14], etc.

AI in healthcare is primarily used by healthcare professionals, but there is an increasing amount of research on this topic [15]. Our goal is to develop a tool that helps family caregivers manage their work-life balance, so that they are able to continue caring for their family members without sacrificing their own work and personal aspirations. In cases of medical emergencies, such as strokes or dementia, patients and their families often find it difficult to access and utilize the support services provided by the German healthcare system. Additionally, there is a lack of coordination and networking structures between different sectors of the healthcare system, leading to a lack of guidance and support for individuals in need of care. In this context, digital case management offers valuable guidance and assistance to patients and their families, helping them navigate their individual care situations. Reeves [16] asserts that digital case management may improve communication and enhance the efficiency of the treatment process for those affected.

Therefore, we have formulated the following research questions:

I. What machine learning-based systems or tools in healthcare can support family caregivers in navigating the healthcare system, identifying the best care options for their family members, and balancing their work and caregiving responsibilities?
II. What type of data is required to train a data-driven tool for initial guidance, and how can it be obtained?
III. What is the underlying machine learning challenge and how can this challenge be addressed in the context of a care situation?
IV. How accurately can the data-driven tool predict the next steps in the care process for a patient during the initial phase, based on answers to a questionnaire completed by the family caregiver, given that the training dataset is familiar with similar care situations?

## 2. Materials and Methods

Machine learning can support family caregivers in navigating the healthcare system, identifying the best care options for their family members, and balancing their work and caregiving responsibilities. Examples include recommender systems [17,18], health management tools [19], and decision support systems [20].

Recommender systems assist family caregivers in finding care options tailored to their family members’ needs by considering factors such as medical conditions, preferences, and location. They provide recommendations for care facilities, services, and providers that are appropriate for the individual’s situation. Health management tools, such as apps, help family caregivers in managing the health and care needs of their family members. These tools provide real-time information on vital signs, adherence to medication, and other health factors, empowering caregivers to spot possible issues and take timely action. Decision support systems facilitate family caregivers in making well-informed decisions about their family members’ care by providing information on different care options, including potential risks and benefits, in addition to guidance on how to pick the best option. They can also assist caregivers in assessing the suitability of different care providers.

We chose to create a recommendation system for the initial stage of a care scenario because it offers valuable guidance to family caregivers who are overwhelmed. Particularly, caregivers who are trying to balance work and caregiving obligations may not have the time or resources to spend hours scouring the web and weighing up different care options. A recommendation system provides them with personalized advice rapidly and effortlessly, saving them time and effort and allowing them to concentrate on their work and caregiving duties. This is especially beneficial for caregivers who are dealing with complex medical conditions or other challenging situations in their lives.

### 2.1. Concept of Digital Case Manager

**Figure 1:**
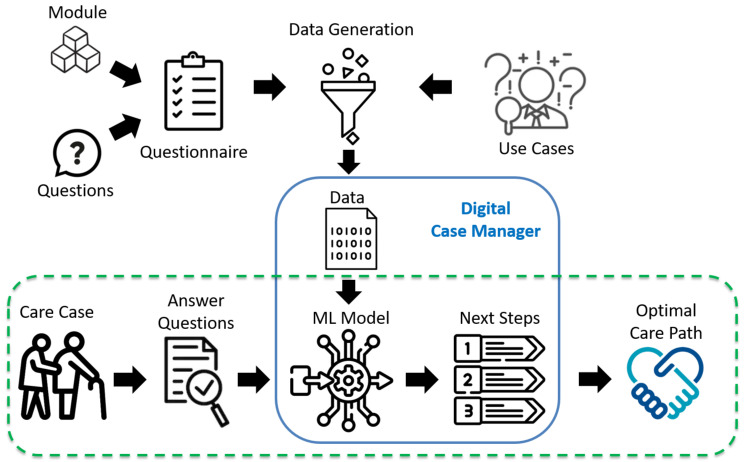
Concept of Digital Case Manager.

The development process for the Digital Case Manager is schematically depicted in Figure 1. The figure is divided into two main areas: the generation of data, which is necessary for creating a machine learning model (ML model), and the creation and application of the model itself. The creation of the ML model and its use are depicted in the lower section of the figure, while the generation of data is depicted in the upper section.

A dashed line indicates the workflow of the Digital Case Manager application. Here, the procedure is laid out, starting with a care case and ending with the application of the ML model and the prediction of the next steps. The Digital Case Manager is highlighted with a solid line and is located in the center. It is built upon the ML model, which discovers the connections between a care situation and the next steps. This enables the model, after receiving the answers from the family caregiver on a questionnaire, to determine the next steps for the specific care situation and to offer initial guidance to those affected. Below is more information on each stage of this process.

### 2.2. Data Generation Concept

**Figure 2:**
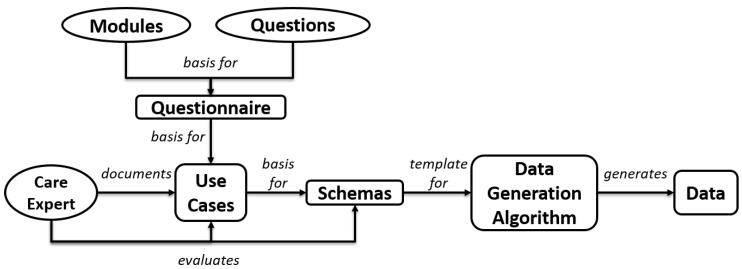
Workflow of Data Generation Process.

Data are essential for the successful implementation of machine learning. In the healthcare sector, it is challenging to obtain real data from sources such as health insurance companies due to data protection regulations. In our scientific work, we encountered this issue and were forced to generate artificial data for our research. It is crucial that the generated data accurately reflect the characteristics and patterns of real-world data so that machine learning models trained on it are able to make accurate predictions and provide useful recommendations. To achieve this, we developed a data generation concept taking into account the various factors that influence care situations and necessary care steps. This concept involves input from professional care experts and aims to provide a comprehensive and realistic representation of care situations and necessary steps. The workflow of the entire data generation process is depicted schematically in Figure 2.

To generate data, a questionnaire is needed that captures the relevant care situation. This allows the physical situation to be translated into data being processed by a computer algorithm. In addition to the questionnaire, we also need use cases that describe and depict specific care situations. These care situations are documented by healthcare experts using the questionnaire, and the recommended care steps are also recorded. Based on the use cases, schemas are constructed that serve as templates, which also allow slight deviations from the original use cases. By means of the schemas, the algorithm generates randomized data, which is a multifaceted and realistic representation. The data generation algorithm is described in depth in Section 2.7.

### 2.3. Questionnaire

The questionnaire is a vital component of our data collection process. It is based on the survey methods of the German Medical Service of the Health Insurance Funds (MDK) [21], which is responsible for providing optimal care within the framework of the socio-medical counseling and assessment tasks for the statutory health and long-term care insurance. The MDK uses a questionnaire, an extended assessment tool, which is intended to avoid reducing the need for care to the need for assistance with everyday tasks. It takes into account impairments of independence and functional limitations in different areas of everyday life [2]. We chose to use the MDK questionnaire as the basis for our survey because it provides objective criteria and comprehensive recording of care situations. Additionally, we included other professional assessment tools from geriatric care, such as the Barthel Index [22] and the questionnaire “Instrumental Activities” according to Lawton/Brody (IADL) [23], based on discussions and literature research. The aim of our questionnaire is to capture the need for help in order to provide the user with suitable recommendations for further care and possible next steps, including references to care and health facilities, service providers, care aids, and barrier-free residential options.

The questionnaire is comprised of seven modules and includes 47 questions with a 4-point Likert scale. These questions are the minimum necessary to cover all important areas of the care situation and thus enable a prediction of the next steps. All of the questions can be found in Appendix A. The use of a Likert scale allows for a standardized and quantitative approach to evaluating the responses, which is important for the accuracy and reliability of the machine learning model.

### 2.4. Modules

The seven modules are the following:

1. Mobility;
2. Cognitive and Communication Skills;
3. Behavioral and Psychological Issues;
4. Self-Care;
5. Managing Health and Therapy Needs;
6. Managing Daily Life and Social Interactions;
7. Organisation of the Care.

The first module, “Mobility”, evaluates whether outside assistance is required for actions like standing up or climbing stairs. There are seven questions on it. The ability to communicate is assessed in the second module, “Cognitive and Communication Skills”, which also looks at other mental impairments. Seven questions are also included in this module. The third module, “Behavioral and Psychological Issues”, contains four questions and focuses on issues like aggressive behavior, anxieties, and depressed moods. Eleven questions make up the fourth module, “Self-Care”, which evaluates the capacity to carry out duties linked to household management and personal hygiene. The fifth module, ”Managing Health and Therapy Needs,“ has eight questions and covers abilities including the capacity to take medication and assess blood sugar on one’s own. The sixth module, ”Managing Daily Life and Social Interactions”, contains four questions that determine the capacity for organizing the day and interacting with others. Six questions make up the last module, “Organisation of the Care”, which focuses on the assistance or care given to the recipient of the assessment.

These modules cover a wide range of important aspects of a care situation, allowing us to collect comprehensive and accurate data that can be used to make recommendations for further care and next steps. They are grounded in best practices and expert techniques from geriatric care, ensuring that the questionnaire is both reliable and effective in assessing the care needs of older individuals.

### 2.5. Questions

To provide further insight into the questionnaire, here is an example of the questions contained in Module 1, “Mobility”:

1. Changing position in bed (e.g., turning and twisting in bed);
2. Holding in the sitting position;
3. Getting up out of the seat;
4. Transferring from the bed, e.g., to the wheelchair;
5. Moving around inside the home;
6. Moving outside the home;
7. Climbing stairs.

These questions are selected and adapted to obtain a thorough understanding about the person’s ability to perform physical activities. They capture the relevant aspects and allow for accurate predictions of the next care steps.

The answer options are as follows:

1. Possible without outside help;
2. Some outside help needed;
3. Outside help predominantly needed;
4. External help completely needed;
5. Not required.

The answer options in the questionnaire allow the person to accurately and thoroughly describe their care situation. This ensures that the recording of the person’s needs and abilities is consistent and follows a standardized way.

### 2.6. Use Cases

The use cases are an essential component of our data generation process, as they provide specific scenarios and context for the questionnaire responses. Based on the ICF (International Classification of Functioning, Disability and Health) from the World Health Organization (WHO) [24], these scenarios focus on stroke, dementia, and caregiving. A total of 28 use cases were carefully crafted by care experts and were selected to provide a representative sample within the limited time frame of our project.

These chronic disease patterns capture the complexity and long-term nature of care situations and aim to depict medical and nursing care as accurately and comprehensively as possible. The case scenarios on the topic of caregiving were deliberately chosen to distinguish them from medically diagnosed diseases in order to crystallize any differences with regard to the care steps. Initially, the Digital Case Manager is only applicable to these specific areas. However, it can be easily expanded to include data from other care situations, such as Parkinson’s disease, as needed.

To expand the data, we developed schemas based on the use cases that serve as templates for our data generation algorithm. These schemas allow for slight variations from the original 28 use cases, while still leading to the same next steps in the care process. The allowed variations were carefully reviewed by care experts to ensure their realism and relevance. The inclusion of such variations in the generated data allows us to capture the diversity of care situations more effectively. By allowing multiple answer options for a given question, even in similar care situations, we are able to reflect the complexity and uniqueness of each individual case. This enhances the quality and realism of the generated data and helps improve the learning of the ML model. Despite the initial focus on certain areas of care, our approach allows us to generate a range of complex care scenarios and enrich the ML model’s knowledge and capabilities.

### 2.7. Data Generation Algorithm

The use cases and their schemas described so far do not provide enough information for machine learning purposes. To properly train and evaluate a machine learning model, we need a dataset that includes multiple care situations. Each row in this dataset should represent a different care situation and include the answers to the questions from the questionnaire. To create this dataset, we use the schemas as templates and vary the answers to the questionnaire based on the allowed variations specified in the schema. To automate this process, we have developed a data generation Algorithm 1.
**Algorithm 1:** Data Generation Algorithm.**Require:** use cases *U*, schemas *S*, questions *Q*, labels *L*   Set the number of data points to be generated, *n*   Initialize a counter i=0   **while** i<n **do**         Randomly select a use case u∈U         Select a schema s∈S for use case *u*         **for** j=1 to the number of questions, *q* **do**             Randomly select an answer *a* for question $q_{j}$ based on the schema *s* for use case *u*         **end for**         Add the labels $l_{u}$ for use case *u*         Increment the counter: i=i+1   **end while**   **return** Generated data

The algorithm starts by randomly selecting one of the 28 available use cases. It then uses the corresponding schema as a template and runs through all the modules and questions of the questionnaire, randomly selecting one of the options for each question. After all questions have been answered, the algorithm stores the next steps in the last column of the data set. This process is repeated by randomly selecting a new use case and creating a new care situation until the requested number of care situations is reached. Each row of the data set therefore corresponds to one generated care situation.

#### Generated Data

To improve the understanding of the data structure, the generated data are displayed in Table 1.

The dataset is organized in a tabular format, with rows representing individual care situations and columns corresponding to the various features and labels. The features consist of the 47 questions posed to the patient or caregiver, while the labels represent the next steps or recommendations for care. To provide a concrete example, consider the first row of the dataset, which pertains to a care situation involving stroke. The first two columns pertain to the “Mobility” module and ask whether the patient requires assistance with tasks such as changing position in bed or holding in a sitting position. This pattern is repeated for all 47 questions. The final column contains the labels, which may include recommendations for outpatient care, care and accompaniment, speech therapy, occupational therapy, household help, and medication. A list of all of the next steps used can be found in Appendix B. From a machine learning perspective, the columns comprising the questions and labels represent the inputs used to train a model. The goal of such a model would be to predict the appropriate next steps for a given care situation based on the patient’s responses to the questions.

## 3. Results

**Figure 3:**
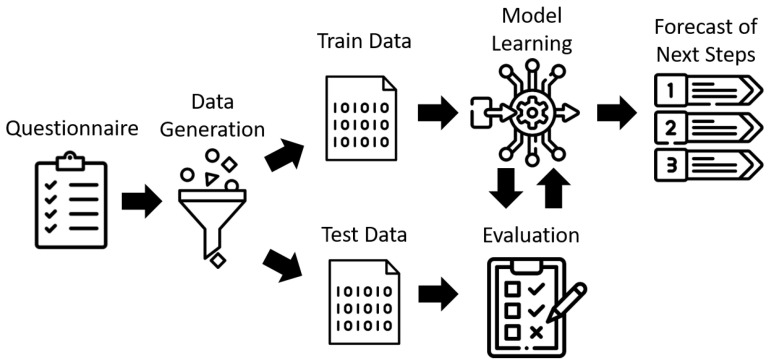
Concept of Machine Learning Model.

The underlying problem of determining the appropriate steps to take in a given care situation can be represented as a multi-label classification task in machine learning. This means that multiple classes (e.g., ergotherapy, medication) can be assigned to a single instance (i.e., care situation) in the Digital Case Manager. Multi-label classification is a type of multi-task learning, a subfield of machine learning where multiple tasks are solved simultaneously [25]. It involves using several binary classifiers to handle individual classes and is similar to multi-class classification, where only one class can be assigned to an instance. However, in multi-label classification, multiple classes per instance are allowed. Multi-label classification is often used to categorize texts or multimedia resources [26]. For example, a newspaper article may be assigned to multiple departments such as politics, economics, and sports. Mathematically, multi-label classification involves finding a model that assigns input data to the appropriate labels (next steps in care). Each label is assigned a value of 0 or 1, with 0 indicating that the label does not apply and 1 indicating that it does. The goal of the machine learning model in this context is to assign the appropriate labels (next steps in care) to each care situation described by the features (answers to the questionnaire). The concept for creating this model is illustrated in Figure 3.

The data generated from the questionnaires were used as input for the model learning algorithm, which is based on the Random Forest Classifier [27]. The Random Forest Classifier is an ensemble learning method that combines several decision trees to make predictions. It was chosen for this particular problem because it is effective for multi-label classification tasks, as demonstrated by Madjarev et al. [28]. However, more advanced machine learning techniques such as neural networks or deep learning algorithms were not applicable in this case because they require large amounts of data to learn patterns and relationships accurately [29]. With a small dataset like ours, the predictions made by these algorithms might not be reliable or accurate. In addition, deep learning algorithms require complex architectures with many layers to learn complex patterns, which makes them less suitable for small datasets. Therefore, we decided to use a simpler machine learning algorithm like the Random Forest Classifier, which can still provide good performance with a limited amount of data. The model learning algorithm is outlined in pseudo code in Algorithm 2.
**Algorithm 2:** Model Learning Algorithm.**Require:** data *D***Ensure:** model *M*    Transform the labels of each row of the data into a list *L*    Convert the answers to the questions into integers *I*    Split the data *D* into training $D_{\mathrm{train}}$ and test sets $D_{\mathrm{train}}$    Train a Multi-Output Classifier MOC using a RandomForestClassifier RFC with $D_{\mathrm{train}}$ as input    Set the number of estimators in RFC to 10    **return** $M=MOC(RFC,n_{\mathrm{estimators}}=10)$

The labels are first transformed from a string to a list on an individual basis. This allows us to approach each step as a separate classification problem, which is the prerequisite if we want to apply the Multi-Output Classifier for prediction. The responses to the questions are also converted into integers in order to simplify processing and the application. Next, training data and test data are separated. This enables us to evaluate the model’s capacity to generalize to novel, unobserved data and produce reliable forecasts on that data. The Multi-Output Classifier is a machine learning model that consists of multiple binary classifiers, each responsible for predicting a specific label or target. In this case, we employ the Random Forest Classifier as the binary classifier and set the hyperparameter for the number of decision trees per Random Forest to 10 to balance computational efficiency and accuracy. To train the model, we utilize a set of labeled data known as the training data. Once the model is trained, it can make predictions on new input data, which includes answers to questions and other relevant information. The model employs the trained binary classifiers to predict the values of each target label, and these predictions can determine the next steps in the process.

### 3.1. Evaluation

Before using the model for prediction, it is important to evaluate its performance. To do this, we use unseen test data, which are not used for training the model. The test data are already in the correct format for the model, as shown in Algorithm 2. The evaluation involves comparing the model’s predictions on the test data to the known ground truth labels. This allows us to measure the model’s accuracy and determine how well it generalizes to new, unseen data. If the model performs well on the test data, we can have confidence in its ability to make accurate predictions on other similar data. We evaluate the model by comparing performance for three different data sets to examine the impact of insufficient data on model performance: Dataset 100, which contains 100 generated care situations; Dataset 500, which contains 500 generated care situations; and Dataset 1000, which contains 1000 generated care situations. For each dataset, the data are separated into training and test sets using a ratio of 70:30. This allows us to train the model on the training data and evaluate its performance on the test data, using the known ground truth labels. By comparing the model’s performance on the different datasets, we are able to investigate the influence of the amount of data on the model’s accuracy and generalization ability.

We have calculated a variety of metrics to evaluate the performance of our model including classical measures such as accuracy, F1-score, and 2-fold and 10-fold cross-validation, as well as metrics specifically tailored to the task of multi-label classification, such as hamming loss, coverage error, label ranking average precision, and label ranking loss. Accuracy is a commonly used metric that assesses the proportion of predictions made by the model that match the labels assigned by experts for a particular care situation. The F1-score is a metric that balances precision and recall, and is calculated as the harmonic mean of these two values. In the context of multi-label classification, the F1-score is the average F1-score for each label, with the weights of each label determined by the number of samples. This allows us to assess the overall performance of the model across all labels, taking into account both precision and recall. Cross-validation is a resampling technique that is applied to evaluate the performance of a model by training and testing it on different subsets of the data [30]. This method provides more robust estimates of the model’s performance, as it is tested on a diverse set of data rather than just a single training or test set. Hamming loss is a metric specifically designed for multi-label classification tasks [31]. It quantifies the average number of labels that are incorrectly predicted by the model, and is calculated by summing the individual binary cross-entropy losses for each label. This metric ranges from 0 to 1, with 0 representing the optimal outcome. Hamming loss is particularly useful for evaluating the performance of a multi-label classification model in cases where the labels are imbalanced or the model predicts a large number of labels. The coverage error is a metric that reflects the average number of labels that must be included in the final prediction in order to correctly identify all true labels [32]. The label ranking average precision score is the average of the correctly assigned labels of the ratio of true labels to the total number of labels with lower scores [28]. The best possible score for this metric is 1. The label ranking loss score represents the average number of label sets that are incorrectly ordered given the predictions of the model, weighted by the size of the label set and the number of labels not included in the label set [32]. The optimal score for this metric is 0.

To compute these metrics, the ML model was firstly learned by using the training data. Then, the test data are used as new input to create predictions of the next steps by the model. These predictions were compared with the actual steps, which were determined by care experts. The results of this evaluation are presented in Table 2.

The model trained with Dataset 100 achieved an accuracy of 80% and a F1-score of 0.9306. The 2-fold cross validation resulted in an accuracy of 56% and 73% for 10-fold cross validation. The model also had a hamming loss of 0.01764 and a coverage error of 6.06. The label ranking average precision score was 0.9209 and the label ranking loss was 0.0648. The model trained with Dataset 500 performed significantly better, with an accuracy of 99.33% and a F1-score of 0.9993. The 2-fold and 10-fold cross validation also showed improved performance, with an accuracy of 97.78% and 99.2%. The model’s hamming loss was 0.00039 and its coverage error was 4.85. The label ranking average precision score increased to 0.9993 and the label ranking loss decreased to 0.0011. The improvement from Dataset 1000 to Dataset 500 was not as significant as the improvement from Dataset 100 to Dataset 500. The model trained with Dataset 1000 had an accuracy of 99.33% and a F1-score of 0.9993. The 2-fold and 10-fold cross validation showed an accuracy of 99.7% and 99.8%. The model also had a hamming loss of 0.00039 and a coverage error of 4.94. The label ranking average precision score and label ranking loss were the same as the model trained with Dataset 500, at 0.9993 and 0.0011, respectively.

These findings indicate that the models developed using 500 or 1000 generated care situations are effective and appropriate for the task at hand. The model chosen for the Digital Case Manager’s implementation was the one that performed the best in the 2-fold and 10-fold cross validation after being trained with 1000 generated care situations. The model’s strong performance in the label ranking metrics, high accuracy, F1-score, low hamming loss, and coverage error support this decision. These outcomes demonstrate the effectiveness of using machine learning to predict the next steps in healthcare scenarios.

### 3.2. Proof-of-Concept

**Figure 4:**
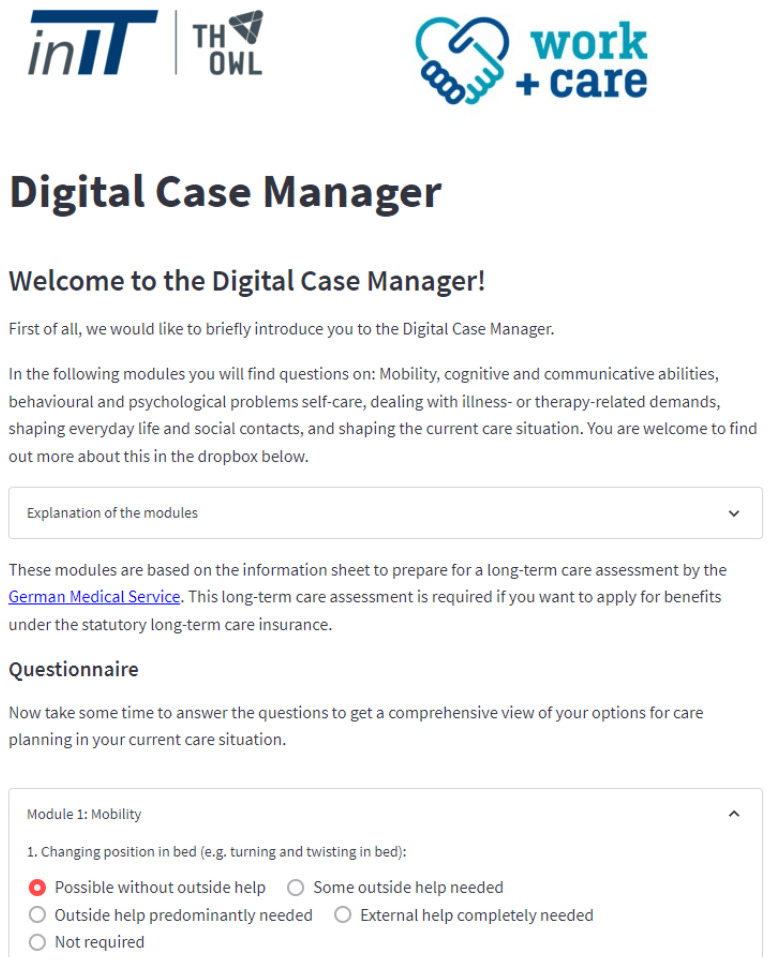
Proof-of-concept implementation.

A proof-of-concept implementation of the Digital Case Manager was developed as part of the research project work and care. This implementation was done in Python 3 using the sklearn and streamlit libraries [33,34,35]. The demonstrator was created for testing and evaluation purposes, including evaluating user comprehensibility in the future. The Digital Case Manager can be accessed through the internet and a web browser and is hosted on the cloud. An example of the Digital Case Manager’s start page is shown in Figure 4.

The prototype of the Digital Case Manager starts with an introduction that explains how to use the tool and what it is for. This is followed by a closed window, which can be opened with the plus symbol in the upper right corner. Here is detailed information on the individual modules of the questionnaire. Next comes the section with the seven modules and their questions. For each question there are answer options that can be selected by means of a radio button. After the question area comes the analysis area. Here is a button called “Start Analysis”, which the user has to press to start the prediction of the next care steps. After the model has determined the next steps, they are listed at the bottom of the page.

In a typical use case of the Digital Case Manager, a healthcare situation arises where the user (the family caregiver) feels overwhelmed and needs initial guidance on the next steps to take. To provide this guidance, the user completes the questionnaire and initiates the prediction of the next steps using the machine learning model. The model calculates the next steps in care and displays them to the user. The user can then click on specific care steps, such as ergotherapy, and be directed to service providers who offer that service in their local area. This creates an optimal care path that allows the user to access the help they need quickly and reliably, around the clock.

In this evaluation we focus only on the technical aspects of the Digital Case Manager, such as development and implementation. An evaluation with family caregivers about the user experience is planned and we will report findings in a future publication.

## 4. Conclusions

The Digital Case Manager demonstrated its potential, particularly in the use cases outlined in Section 2.6, although it may not be as accurate in other situations or contexts. It was specifically developed to help family caregivers who are suddenly faced with a caregiving situation and need guidance. The Digital Case Manager uses a questionnaire-based approach that allows caregivers to intuitively answer questions about the care situation. For the initial phase, this enables the integrated machine learning model to provide accurate recommendations for the next steps in the care process. However, professional support is needed for the further steps in care, as the Digital Case Manager only serves as an initial orientation. In this way, the Digital Case Manager provides quick and convenient support for family caregivers facing a challenging or difficult situation, although it may not be suitable for all caregiving situations.

### 4.1. Findings

Regarding the question: *What machine learning-based systems or tools in healthcare can support family caregivers in navigating the healthcare system, identifying the best care options for their family members, and balancing their work and caregiving responsibilities?* Machine learning can be a valuable resource for family caregivers, as our literature research has shown. We identified three different kinds of systems that are able to assist caregivers in their duties: recommender systems, health management tools, and decision support systems. Recommender systems use algorithms to provide personalized recommendations for care facilities, services, and providers that meet the specific needs of family members. Health management tools help caregivers track and monitor the health of their family members, providing real-time updates and alerts for potential issues. Decision support systems offer caregivers valuable information about various care options, supporting informed decision-making. In our research, we focused further on a recommender system, which we found to be particularly promising and effective in supporting family caregivers with regard to balancing work and care.

Regarding the question: *What type of data is required to train a data-driven tool for initial guidance and how can it be obtained?* High-quality data that reflect the characteristics and patterns of real-world scenarios are necessary in order to create a trustworthy data-driven tool for giving initial guidance in care settings. Unfortunately, acquiring such information can be tough. In order to overcome this difficulty, we developed a model for data generation that includes input from professionals in the field of care and seeks to provide a realistic representation of care needs and the steps required to address them.

Regarding the question: *What is the underlying machine learning challenge and how can this challenge be addressed in the context of a care situation?* The machine learning challenge presented in the context of care situations is a multi-label classification problem, in which multiple classes (next steps) can be assigned to a given instance (care situation). The multiple classes correspond to the next steps in care and one instance corresponds to one care situation. The answers to the 47 questions are accordingly the features. To address this challenge, we employed a multi-label classifier based on the Random Forest algorithm to learn the relationship between the care situation, which are described by the features and the next steps of care.

Regarding the question: *How accurately can the data-driven tool predict the next steps in the care process for a patient during the initial phase, based on answers to a questionnaire completed by the family caregiver, given that the training dataset is familiar with similar care situations?* Our research revealed that the machine learning approach we developed had high accuracy when trained with Dataset 1000, with an accuracy of 99.33% and a F1-score of 0.9993. Cross-validation results demonstrated a high level of accuracy, with the 2-fold cross-validation achieving 99.7% accuracy and the 10-fold cross-validation achieving 99.8% accuracy. The model also had a low hamming loss of 0.00039, implying its error in predicting the next steps in the care process was very low. These results demonstrate the effectiveness of the machine learning approach in accurately predicting the next steps in the initial phase of a patient’s care process, provided that a similar care situation was included in the training dataset.

### 4.2. Strengths and Weaknesses

The Digital Case Manager is a tool that provides advice and support to assist family caregivers in navigating the complex healthcare system and making informed decisions about care options for their family members. It offers valuable information about existing support structures and facilitates the identification of appropriate care options, leading to cost and time efficiencies. The Digital Case Manager streamlines the caregiving process, helping family caregivers remain in the workforce by freeing up their time and addressing the challenges of demographic change in the Western world. However, it is important to recognize that the Digital Case Manager may not be accurate in all care situations and is just one resource among many that caregivers can utilize. Additionally, while it provides quick and convenient support for family caregivers facing a challenging situation, it is not able to fully replace the support and guidance of a human care manager. So, if in doubt, it is advised to consult a professional.

One limitation is that the tool’s machine learning model was trained using information from only 28 care scenarios based on stroke, dementia, and caregiving. As a result, it might not provide reliable recommendations for other care scenarios. In order to address this issue, more data of other care situations need to be gathered in collaboration with a care facility or health insurance provider. This will not only improve the tool’s data realism, but also help to ensure that the Digital Case Manager is able to provide recommendations for a wider range of care scenarios. Another limitation of the Digital Case Manager is that it does not sufficiently consider the perspectives of patients and the family members who care for them. In the future, we intend to address this by evaluating actual care scenarios with the patient and the family caregiver present. Their knowledge and experience will be added to improve the tool further. Our preliminary evaluations, conducted with both family caregivers and professional experts, suggest that the Digital Case Manager has the potential to be a valuable tool, but further development and field testing are required. The tool’s current version was specifically designed for family caregivers who are not familiar with the medical field as the target group. To increase the effectiveness of the Digital Case Manager for professional healthcare personnel, it will need to be customized to their specific needs and preferences.

### 4.3. Outlook

In the future, an idea is to integrate the Digital Case Manager into a health platform and to connect the individual steps recommended by the model to the services and resources offered by providers such as pharmacies and care facilities. This would allow users to easily access the services and resources they need to follow the recommended next steps and manage their healthcare more effectively.

We intend to explore additional possible applications for the Digital Case Manager in addition to improving its functionality. Healthcare professionals might employ it, for instance, to simplify their workflows and devote more time providing care to patients. We are aware that the current recommendations are not comprehensive enough to offer assistance to professional caregivers. We plan to develop more precise recommendations in the future that will offer the required level of detail and support to address this issue.

The flexibility of the Digital Case Manager to adapt to particular addressing scenarios, such post-stroke care, may serve as a further starting point for future development to provide more tailored assistance. Here, the tool would have the potential to increase the standard of procedures and care while streamlining patient care throughout the whole healthcare system. As a result, stroke victims may receive better, more efficient care in the long run, and the healthcare system may have an easier time allocating resources for its medical and nursing services.

## Figures and Tables

**Table 1:** Structure of the generated dataset.

Changing Position in Bed (e.g., Turning and Twisting in Bed)	Holding in the Sitting Position	…	Professional Service Providers Such as Home Care Services or Residential Care Facilities	Next Steps (Labels)
Possible without outside help	Possible without outside help	…	Yes	Ambulatory care
				Care & accompaniment
				Speech therapy
				Occupational therapy
				Home help
				Medication
Outside help predominantly needed	Outside help predominantly needed	…	No	Ambulatory care
				Care & accompaniment
				Physiotherapy
				Occupational therapy
				Speech therapy
				Assistive devices
				Medication
				Self-help & support options
Some outside help needed	Some outside help needed	…	No	Ambulatory care
				Care & accompaniment
				Physiotherapy
				Occupational therapy
				Assistive devices
				Medication
…	…	…	…	…
Outside help predominantly needed	External help completely needed	…	Yes	Inpatient care

**Table 2:** Evaluation of model.

Metrics	Dataset 100	Dataset 500	Dataset 1000
Accuracy	80.00%	99.33%	99.33%
F1-Score	0.9306	0.9993	0.9993
2-Fold CV	56%	97.78%	99.7%
10-Fold CV	73%	99.2%	99.8%
Hamming loss	0.01764	0.00039	0.00039
Coverage error	6.06	4.85	4.94
Label ranking average precision	0.9209	0.9993	0.9993
Label ranking loss	0.0648	0.0011	0.0011

## Data Availability

Data available on request.

## Appendix A

### Appendix A.1. Questions

#### Appendix A.1.1. Module 1 Mobility

1. Changing position in bed (e.g., turning and twisting in bed)
2. Holding in the sitting position
3. Getting up out of the seat
4. Transferring from the bed, e.g., to the wheelchair
5. Moving around inside the home
6. Moving outside the home
7. Climbing stairs

#### Appendix A.1.2. Module 2 Cognitive and Communication Skills

8. Recognition of familiar people
9. Awareness of time and date
10. Remembrance of past events (e.g., family celebrations, trips)
11. Can dangers be assessed (cooker switched off?)
12. Communication of needs (e.g., hunger or thirst)
13. Participation in conversations
14. Understanding of requests

#### Appendix A.1.3. Module 3 Behavioral and Psychological Issues

15. Aggressive or defensive behavior
16. Lethargy
17. Anxiety
18. Sad mood

#### Appendix A.1.4. Module 4 Self-Care

19. Brushing, shaving, and brushing teeth
20. Washing, bathing, and showering
21. Dressing and undressing
22. Toilet use
23. Chopping food, opening bottles
24. Eating and drinking
25. Food preparation
26. Shopping
27. Cleaning of apartment or house
28. Maintenance of apartment or house
29. Gardening

#### Appendix A.1.5. Module 5 Managing Health and Therapy Needs

30. Provision and/or administration of medications
31. Putting on compression stockings
32. Administration of injections
33. Changing bandages/ wound care
34. Physical therapy exercises
35. Visits to the doctor or other medical facilities
36. Following a diet
37. Measurement of blood pressure or blood sugar levels, etc.

#### Appendix A.1.6. Module 6 Managing Daily Life and Social Interactions

38. Personal occupation (e.g., reading, listening to music)
39. Design of the daily routine (e.g., breakfast, lunch, and dinner)
40. Organization of activities (e.g., walk/ride with acquaintances/relatives)
41. Personal interaction with other people (e.g., personal conversation, phone call, WhatsApp, email)

#### Appendix A.1.7. Module 7 Organisation of the Care

42. Spouse or life partner
43. Parents or children
44. Close relatives such as siblings or nieces/nephews
45. Acquaintances or friends
46. Neighbors or volunteers
47. Professional service providers such as home care services or residential care facilities

## Appendix B

### Next Steps

- Age-appropriate living at home;
- Ambulatory care;
- Consultation with a doctor;
- Care and accompaniment;
- Shopping assistance;
- Occupational therapy;
- Nutrition support;
- Household help;
- Assistive devices;
- Speech therapy;
- Medication;
- Mobility and movement;
- Physical therapy;
- Psychotherapy;
- Self-help and support options;
- Inpatient care (hospitalization);
- No further action needed.

## References

[1] Sozialgesetzbuch XI (1994): Elftes Buch (XI) (2021). Soziale Pflegeversicherung (Artikel 1 des Gesetzes vom 26. Mai 1994, BGBl. I S. 1014). Zuletzt Geändert Durch Art. 2d G v. 28.6.2022 I 938. https://www.gesetze-im-internet.de/sgb_11/BJNR101500994.html.

[2] Glaeske G. (2020). Demenzreport 2020.

[3] Glauner P. (2021). Artificial Intelligence in Healthcare: Foundations, Opportunities and Challenges. Digitalization in Healthcare.

[4] Qomariah D.U.N., Tjandrasa H., Fatichah C. Classification of diabetic retinopathy and normal retinal images using CNN and SVM. Proceedings of the 2019 12th International Conference on Information & Communication Technology and System (ICTS).

[5] Tigga N.P., Garg S. (2020). Prediction of type 2 diabetes using machine learning classification methods. Procedia Comput. Sci..

[6] Ortega J.H.J.C., Resureccion M.R., Natividad L.R.Q., Bantug E.T., Lagman A.C., Lopez S.R. (2020). An analysis of classification of breast cancer dataset using J48 algorithm. Int. J. Adv. Trends Comput. Sci. Eng..

[7] Mahajan S., Bangar G., Kulkarni N. (2020). Machine Learning Algorithms for Classification of Various Stages of Alzheimer’s Disease: A review. Mach. Learn..

[8] Qin J., Chen L., Liu Y., Liu C., Feng C., Chen B. (2019). A machine learning methodology for diagnosing chronic kidney disease. IEEE Access.

[9] Bhaskar N., Manikandan S. (2019). A deep-learning-based system for automated sensing of chronic kidney disease. IEEE Sens. Lett..

[10] Shankar K., Lakshmanaprabu S., Gupta D., Maseleno A., De Albuquerque V.H.C. (2020). Optimal feature-based multi-kernel SVM approach for thyroid disease classification. J. Supercomput..

[11] Kang J., Ullah Z., Gwak J. (2021). Mri-based brain tumor classification using ensemble of deep features and machine learning classifiers. Sensors.

[12] Roberts M., Driggs D., Thorpe M., Gilbey J., Yeung M., Ursprung S., Aviles-Rivero A.I., Etmann C., McCague C., Beer L. (2021). Common pitfalls and recommendations for using machine learning to detect and prognosticate for COVID-19 using chest radiographs and CT scans. Nat. Mach. Intell..

[13] Schiaffino S., Codari M., Cozzi A., Albano D., Alì M., Arioli R., Avola E., Bnà C., Cariati M., Carriero S. (2021). Machine learning to predict in-hospital mortality in covid-19 patients using computed tomography-derived pulmonary and vascular features. J. Pers. Med..

[14] Kebir S., Schmidt T., Weber M., Lazaridis L., Galldiks N., Langen K.J., Kleinschnitz C., Hattingen E., Herrlinger U., Lohmann P. (2020). A preliminary study on machine learning-based evaluation of static and dynamic FET-PET for the detection of pseudoprogression in patients with IDH-wildtype glioblastoma. Cancers.

[15] Pfannstiel M.A. (2022). Künstliche Intelligenz im Gesundheitswesen-Entwicklungen, Beispiele und Perspektiven.

[16] Reeves C. (2020). Casemanagement, digital. Health & Care Management-Digital Health Lexikon.

[17] De Croon R., Van Houdt L., Htun N., Štiglic G., Vanden Abeele V., Verbert K. (2021). Health Recommender Systems: Systematic Review. J. Med. Internet Res..

[18] Tran T., Felfernig A., Trattner C. (2021). Recommender systems in the healthcare domain: State-of-the-art and research issues. J. Intell. Inf. Syst..

[19] Ruyobeza B., Grobbelaar S., Botha A. (2022). Hurdles to developing and scaling remote patients’ health management tools and systems: A scoping review. Syst. Rev..

[20] Musen M.A., Middleton B., Greenes R.A., Shortliffe E.H., Cimino J.J. (2021). Clinical Decision-Support Systems. Biomedical Informatics: Computer Applications in Health Care and Biomedicine.

[21] Medizinischer Dienst Der Krankenversicherung Nordrhein (2021). Der Auskunftsbogen zur Vorbereitung auf das Gespräch mit dem Medizinischen Dienst. https://www.md-nordrhein.de/.

[22] Mahoney F.I., Barthel D.W. (1965). Functional Evaluation: The Barthel Index. Md. State Med. J..

[23] Lawton M.P., Brody E.M. (1969). Assessment of older people: Self-maintaining and instrumental activities of daily living. Gerontologist.

[24] (2010). ICF—Internationale Klassifikation der Funktionsfähigkeit, Behinderung und Gesundheit.

[25] Zhang Y., Yang Q. (2018). An overview of multi-task learning. Natl. Sci. Rev..

[26] Herrera F., Charte F., Rivera A.J., del Jesus M.J. (2016). Multilabel Classification. Multilabel Classification: Problem Analysis, Metrics and Techniques.

[27] Ho T.K. Random decision forests. Proceedings of the 3rd International Conference on Document Analysis and Recognition.

[28] Madjarov G., Kocev D., Gjorgjevikj D., Džeroski S. (2012). An extensive experimental comparison of methods for multi-label learning. Pattern Recognit..

[29] Alzubaidi L., Zhang J., Humaidi A.J., Al-Dujaili A., Duan Y., Al-Shamma O., Santamaría J., Fadhel M.A., Al-Amidie M., Farhan L. (2021). Review of deep learning: Concepts, CNN architectures, challenges, applications, future directions. J. Big Data.

[30] James G., Witten D., Hastie T., Tibshirani R. (2013). An Introduction to Statistical Learning.

[31] Schapire R.E., Singer Y. Improved boosting algorithms using confidence-rated predictions. Proceedings of the Eleventh Annual Conference on COMPUTATIONAL Learning Theory.

[32] Tsoumakas G., Katakis I., Vlahavas I. (2009). Mining multi-label data. Data Mining and Knowledge Discovery Handbook.

[33] Van Rossum G., Drake F.L. (2009). Python 3 Reference Manual.

[34] Pedregosa F., Varoquaux G., Gramfort A., Michel V., Thirion B., Grisel O., Blondel M., Prettenhofer P., Weiss R., Dubourg V. (2011). Scikit-learn: Machine Learning in Python. J. Mach. Learn. Res..

[35] Streamlit Inc Streamlit Documentation. https://docs.streamlit.io/.

